# Supplementary material for: Multi‐dataset identification of innovative feature genes and molecular mechanisms in keratoconus
Source: J Cell Mol Med. 2024 Sep 19;28(18):e70079. doi: 10.1111/jcmm.70079 (PMC11412914; doi:10.1111/jcmm.70079)
Supplement: Supplementary file 2 — Material S2. [file JCMM-28-e70079-s002.zip › 7.GSEA analysis/2.ATOH7/KEGG_GLYCOSYLPHOSPHATIDYLINOSITOL_GPI_ANCHOR_BIOSYNTHESIS.html]

Details for gene set KEGG\_GLYCOSYLPHOSPHATIDYLINOSITOL\_GPI\_ANCHOR\_BIOSYNTHESIS[GSEA]

|  || Dataset | ATOH7.group.cls#h\_versus\_l |
| Phenotype | group.cls#h\_versus\_l |
| Upregulated in class | l |
| GeneSet | KEGG\_GLYCOSYLPHOSPHATIDYLINOSITOL\_GPI\_ANCHOR\_BIOSYNTHESIS |
| Enrichment Score (ES) | -0.1989396 |
| Normalized Enrichment Score (NES) | -0.62916744 |
| Nominal p-value | 0.8678414 |
| FDR q-value | 1.0 |
| FWER p-Value | 1.0 |
Table: GSEA Results Summary

  

Fig 1: Enrichment plot: KEGG\_GLYCOSYLPHOSPHATIDYLINOSITOL\_GPI\_ANCHOR\_BIOSYNTHESIS      
 Profile of the Running ES Score & Positions of GeneSet Members on the Rank Ordered List

  

| SYMBOL | TITLE | RANK IN GENE LIST | RANK METRIC SCORE | RUNNING ES | CORE ENRICHMENT || 1 | PIGZ | na | 6904 | 0.145 | -0.1163 | Yes |
| 2 | PIGB | na | 8589 | 0.107 | -0.1041 | Yes |
| 3 | PIGV | na | 9393 | 0.092 | -0.0749 | Yes |
| 4 | PIGA | na | 10472 | 0.074 | -0.0638 | Yes |
| 5 | PIGO | na | 10493 | 0.074 | -0.0224 | Yes |
| 6 | PIGT | na | 10892 | 0.068 | 0.0046 | Yes |
| 7 | PIGC | na | 10960 | 0.067 | 0.0405 | Yes |
| 8 | PIGH | na | 11238 | 0.063 | 0.0682 | Yes |
| 9 | DPM2 | na | 11381 | 0.061 | 0.0987 | Yes |
| 10 | PIGU | na | 11564 | 0.058 | 0.1264 | Yes |
| 11 | PIGQ | na | 12825 | 0.042 | 0.1142 | Yes |
| 12 | PIGF | na | 12859 | 0.042 | 0.1371 | Yes |
| 13 | PIGX | na | 13177 | 0.038 | 0.1498 | Yes |
| 14 | PIGP | na | 13809 | 0.032 | 0.1501 | Yes |
| 15 | PIGG | na | 14741 | 0.023 | 0.1365 | No |
| 16 | PIGK | na | 15311 | 0.018 | 0.1305 | No |
| 17 | PIGN | na | 16486 | 0.009 | 0.1017 | No |
| 18 | PIGS | na | 16974 | 0.005 | 0.0904 | No |
| 19 | GPAA1 | na | 17315 | 0.002 | 0.0815 | No |
| 20 | PIGY | na | 19644 | -0.018 | 0.0249 | No |
| 21 | PIGW | na | 21347 | -0.035 | -0.0039 | No |
| 22 | PIGM | na | 26619 | -0.117 | -0.0891 | No |
| 23 | GPLD1 | na | 27461 | -0.137 | -0.0352 | No |
| 24 | PIGL | na | 28645 | -0.168 | 0.0265 | No |
| 25 | PGAP1 | na | 31353 | -0.261 | 0.0973 | No |
Table: GSEA details [plain text format]

  

Fig 2: KEGG\_GLYCOSYLPHOSPHATIDYLINOSITOL\_GPI\_ANCHOR\_BIOSYNTHESIS      
 Blue-Pink O' Gram in the Space of the Analyzed GeneSet

  

Fig 3: KEGG\_GLYCOSYLPHOSPHATIDYLINOSITOL\_GPI\_ANCHOR\_BIOSYNTHESIS: Random ES distribution      
 Gene set null distribution of ES for **KEGG\_GLYCOSYLPHOSPHATIDYLINOSITOL\_GPI\_ANCHOR\_BIOSYNTHESIS**
